# Supplementary material for: Strong Gene Flow Undermines Local Adaptations in a Host Parasite System
Source: Insects. 2020 Sep 1;11(9):585. doi: 10.3390/insects11090585 (PMC7564341; doi:10.3390/insects11090585)
Supplement: Supplementary file 1 [file insects-11-00585-s001.zip › Sepp_et al_supplement Figures S1-S2.docx]

# Supplementary material: Figures S1-S2

# Article title: Strong gene flow undermines local adaptations in a host parasite system

**Authors:** Perttu Seppä, Mariaelena Bonelli, Simon Dupont, Sanja Hakala, Anne-Geneviève Bagnères and Maria Cristina Lorenzi

Correspondence: perttu.seppa@helsinki.fi


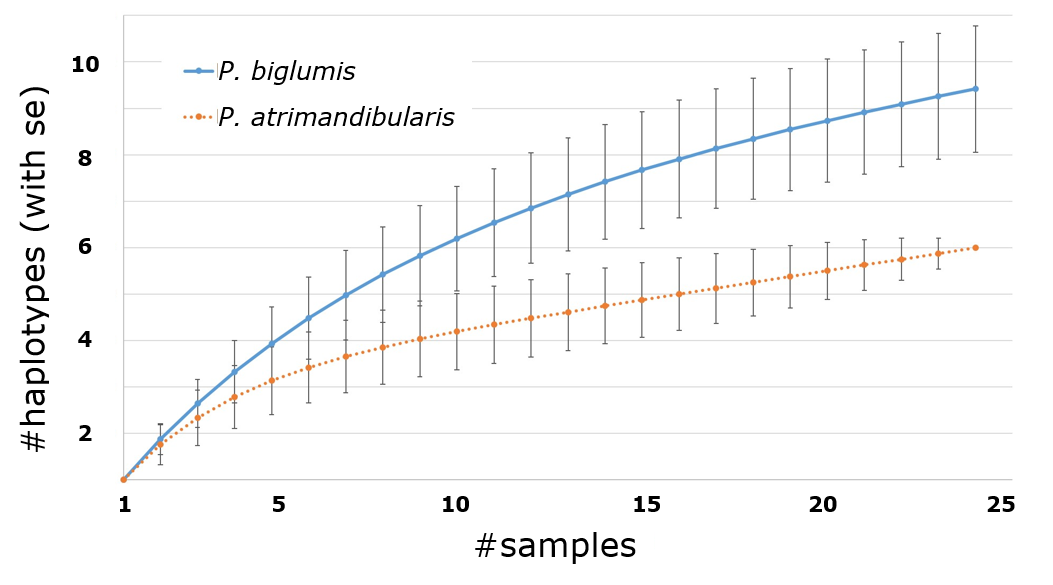


**Figure S1.** Rarefaction curves showing the changes in expected haplotype count (with se) in random subsamples of different sample sizes for each study species.


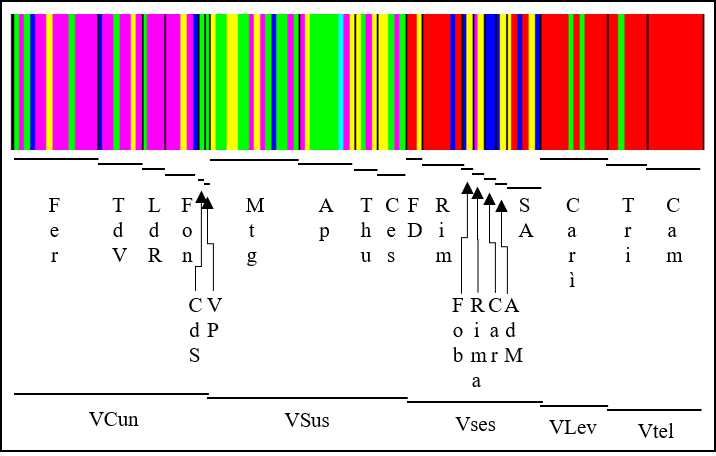


**Figure S2.** Bayesian clustering of mitochondrial sequences of *P. biglumis*. Populations [above] and regions [below] are coded as in Table S1 and ordered geographically from south-west to east.
