# Supplementary material for: Strong Gene Flow Undermines Local Adaptations in a Host Parasite System
Source: Insects. 2020 Sep 1;11(9):585. doi: 10.3390/insects11090585 (PMC7564341; doi:10.3390/insects11090585)
Supplement: Supplementary file 1 [file insects-11-00585-s001.zip › Sepp_et al_supplement Molecular methods.docx]

# **Supplementary material: Molecular methods**

# **Article t**itle: **Strong gene flow undermines local adaptations in a host parasite system**

**Authors:** Perttu Seppä, Mariaelena Bonelli, Simon Dupont, Sanja Hakala, Anne-Geneviève Bagnères and Maria Cristina Lorenzi

Correspondence: perttu.seppa@helsinki.fi

### DNA extraction

Genomic DNA was extracted and purified from one leg with Macherey-Nagel NucleoSpin® Tissue kit (DNA microsatellites, AFLP) or Wizard® Genomic DNA Purification Kit (Promega, sequencing samples), according to manufacturer’s instructions.

### DNA microsatellites

Six DNA microsatellite loci previously developed for *P. dominula* (Pdom1, Pdom7, Pdom25, Pdom140, Pdom121, Pdom139; Henshaw 2000) were amplified in touchdown PCR. Primers were pooled in 2 multiplex panels. Primers were added to 5 μl of Type-it Microsatellite PCR solution (Qiagen) and 1 μl of undiluted sample DNA, and the reaction volume was adjusted to 10 μl with MilliQ water. PCR products were analyzed in a 3730 ABI 3730 DNA analyzer and the resulting electropherograms were interpreted by using GeneMapper 5.0 (Applied Biosystems).

### Amplified fragment length polymorphism (AFLP)

All samples were analysed for AFLP polymorphisms and in addition, 31 and 2 samples (10%) were replicated in *P. biglumis* and *P. atrimandibularis*, respectively, starting from the DNA extraction. Also negative samples were analysed throughout the protocol. AFLP fragments were generated following standard protocols modified from Vos et al. (1995). DNA (150 ng) was digested with restriction enzymes EcoRI (5 U) and MseI (2 U) (NEB) in a total volume of 10 μL for 17 h at 37 °C. Ligation of adaptors E (2 pmol) and M (20 pmol) to the ends of the DNA fragments was made with 0,6 μL T-4 ligase (NEB) in total volume 12,5 μL for 3 h at 37 °C and 1 h at 16 °C.

For the pre-amplification by PCR, the samples were diluted (10x) and 5 μL of the dilution was used in the reaction. The E-primer did not have a selective base and M-primer had C as the selective base. The pre-amplification reaction was carried out using 20 cycles (94 °C, 30 s; 56 °C, 30 s; 72 °C, 60 s). The pre-amplified product was diluted (10×) and 2.5 μL was used for selective amplification. Six primer combinations were employed: 1: EcoRI-TA and MseI-CTT; 2: E-TA and M-CAA; 3: E-TA and M-CTA; 4: E-TG and M-CAA; 5: E-TG and M-CAT; 6: E-AG and M-CAA. Amplification was performed with 12 touchdown cycles (94 °C, 30 s; 65 °C reduced by 0.7 °C per cycle, 30 s; 72 °C, 60 s) followed by 23 cycles of the original program (94 °C, 30 s; 56 °C, 30 s; 72 °C, 60 s). The final PCR products were diluted 100 fold and separated by capillary electrophoresis with GeneScanTM 500 ROX (Applied Biosystems) as internal size standard, using an ABI 3730 DNA analyzer (Applied Biosystems).

The data was visualised, checked for failures, normalised (sum of signal) and scored for the presence and absence with Genemapper 5.0 (Applied Biosystems) software using 100-500 bp as the analysis range and 50 RFU as peak amplitude threshold for all primer combinations. Bin width was set as 1. Peaks under 200 RFU were scored as 0 and over 200 RFU as 1.

### Mitochondrial sequences

From each individual, we amplified a 658 bp portion of the COI gene and a 460 bp portion of the 16S gene using the primers LCO1490 (5′-GGTCAACAAATCATAAAGATATTGG-3′) and HCO2198 (5′- TAAACTTCAGGGTGACCAAAAAATCA-3′) (Folmer *et al*. 1994) for COI, and 16S.Sh (5’-AGATTTTAAAAGTCGAACAG-3’) and 16SWb (5’-CACCTGTTTATCAAAAAACAT-3’) (Dowton and Austin, 1994) for 16S. PCR amplifications were performed with HotstarTaq® Master Mix Kit (Qiagen) with an initial denaturation step at 95 °C (15 min) followed by 35 cycles at 94 °C (30 sec), 54 °C (1 min 30 sec), and 72 °C (2 min), and finally by an extension step at 72 °C (10 min). The PCR templates were sequenced on an automated ABI 3730XL sequencer. Both strands of PCR products were sequenced. Sequences were aligned using the ClustalW algorithm (Thompson *et al*. 1994) within the software Geneious® Pro 9.0.5 and corrected manually. Sequences then were deposited in GenBank under the accession numbers XXXX to XXXX.

### References

Dowton M, Austin AD (1994) Molecular phylogeny of the insect order Hymenoptera: Apocritan relationships. Proceedings of the National Academy of Sciences of the United States of America 91:9911-9915

Folmer O, Black M, Hoeh W, Lutz R, Vrijenhoek R (1994) DNA primers for amplification of mitochondrial cytrochrome c oxidase subunit I from diverse metazoan invertebrates. Molecular Marine Biology Biotechnology 3:294-299

Henshaw MT (2000) Microsatellite loci for the social wasp *Polistes dominulus* and their application in other polistine wasps. Mol Ecol 9:2155-2157

Thompson JD, Higgins DG, Gibson TJ (1994) Clustal-w - Improving the sensitivity of progressive multiple sequence alignment through sequence weigthing, position-specific, gap penalties and weight matrix choice. Nucleic Acids Research 22:4673-4680

Vos P, Hogers R, Bleeker M, Reijans M, van de Lee T, Hornes M, Frijters A, Pot J, Peleman J, Kuiper M, Zabeau M 1995. *AFLP: a new technique for DNA fingerprinting*, Nucleic Acids Research, 23: 4407–4414
