# Supplementary material for: Strong Gene Flow Undermines Local Adaptations in a Host Parasite System
Source: Insects. 2020 Sep 1;11(9):585. doi: 10.3390/insects11090585 (PMC7564341; doi:10.3390/insects11090585)
Supplement: Supplementary file 1 [file insects-11-00585-s001.zip › Sepp_et al_supplement Tables S1-S10.docx]

# Supplementary material: Tables S1-S10

# Article title: Strong gene flow undermines local adaptations in a host parasite system

**Authors:** Perttu Seppä, Mariaelena Bonelli, Simon Dupont, Sanja Hakala, Anne-Geneviève Bagnères and Maria Cristina Lorenzi

Correspondence: perttu.seppa@helsinki.fi

**Table S1.** Study populations, location (Lat = latitude; Long = longitude; Alt = altitude), year of sampling (Year) and sample sizes separately for nuclear (n) and mitochondrial (mt) markers.

| **Population** | **Code** | **Location** | | | **Year** | **Sample size** | | | |
| --- | --- | --- | --- | --- | --- | --- | --- | --- | --- |
|  |  | Lat | Long | Alt |  | *P. biglumis* | | *P. atrimandibularis* | |
|  |  |  |  | m a.s.l. |  | n | mt | n | mt |
| *Valli del Cuneese* | VCun |  |  |  |  |  |  |  |  |
| Ferrere | Fer | 44.37 | 6.95 | 1900 | 2011-2013 | 35 | 15 | 11 | 11 |
| Terme di Valdieri | TdV | 44.20 | 7.27 | 1360 | 2011, 2013 | 9 | 8 |  |  |
| Lago della Rovina | LdR | 44.17 | 7.33 | 1530 | 2011 | 4 | 4 |  |  |
| Fondovet | Fon | 44.57 | 7.13 | 1530 | 2011, 2013 | 7 | 6 |  |  |
| Colle di Sampeyre | CdS | 44.35 | 7.1 | 1300 | 2011 | 1 | 1 |  |  |
| Valle Pesio | VP | 44.11 | 7.37 | 1400 | 2011 | 1 | 1 |  |  |
| *Val Susa* | VSus |  |  |  |  |  |  |  |  |
| Montgenèvre | Mtg | 44.92 | 6.72 | 1850 | 2005, 2011-2013 | 48 | 16 | 17 | 8 |
| Alpe Plane | AP | 44.88 | 6.93 | 1900 | 2005, 2011, 2013 | 16 | 10 |  |  |
| Val di Thuras | Thu | 44.9 | 6.82 | 1700 | 2011 | 8 | 4 | 1 | 1 |
| Cesana | Ces | 44.95 | 6.78 | 1370 | 2011, 2013 | 9 | 5 |  |  |
| *Valsesia* | Vses |  |  |  |  |  |  |  |  |
| Frazione Dorf | FD | 45.67 | 7.92 | 1700 | 2011 | 5 | 3 |  |  |
| Rimella | Rim | 45.9 | 8.2 | 1480 | 2011 | 11 | 7 |  |  |
| Fobello | Fob | 45.9 | 8.15 | 1450 | 2011 | 2 | 2 |  |  |
| Rima | Rima | 45.88 | 7.98 | 1430 | 2011 | 2 | 2 |  |  |
| Carcoforo | Car | 45.9 | 8.05 | 1260 | 2011 | 2 | 2 |  |  |
| Alpe di Mera | AdM | 45.73 | 8.08 | 1635 | 2011 | 2 | 2 |  |  |
| Sant’Antonio | SA | 45.8 | 7.92 | 1350 | 2011, 2013 | 9 | 6 |  |  |
| *Val Leventina* | VLev |  |  |  |  |  |  |  |  |
| Carì | Carì | 46.48 | 8.82 | 1700 | 2011, 2013 | 21 | 12 |  |  |
| *Valtellina* | Vtel |  |  |  |  |  |  |  |  |
| Trivigno | Tri | 46.18 | 10.18 | 1700 | 2011, 2013 | 11 | 7 | 3 | 3 |
| Campovecchio | Cam | 46.13 | 10.18 | 1300 | 2011, 2013 | 12 | 10 | 1 | 1 |

**Table S2**. Allele count (*N*_a_), allelic richness (*A*_R_) and expected heterozygosity according to Hardy-Weinberg equilibrium (*H*_E_) in DNA microsatellite loci studied in each population, calculated for populations with sample size n>2. Populations are coded as in Table S1.

| Population |  | Pdom1 | | | Pdom7 | | | Pdom25 | | | Pdom140 | | | Pdom121 | | | Pdom139 | | | Average | | |
| --- | --- | --- | --- | --- | --- | --- | --- | --- | --- | --- | --- | --- | --- | --- | --- | --- | --- | --- | --- | --- | --- | --- |
|  | n | *N*_a_ | *A_R_* | *H*_E_ | *N*_a_ | *A_R_* | *H*_E_ | *N*_a_ | *A_R_* | *H*_E_ | *N*_a_ | *A_R_* | *H*_E_ | *N*_a_ | *A_R_* | *H*_E_ | *N*_a_ | *A_R_* | *H*_E_ | *N*_a_ | *A_R_* | *H*_E_ |
| ***P. biglumis*** |  |  |  |  |  |  |  |  |  |  |  |  |  |  |  |  |  |  |  |  |  |  |
| Fer | 35 | 12 | 4.4 | 0.86 | 8 | 3.2 | 0.69 | 9 | 3.9 | 0.81 | 6 | 2.7 | 0.58 | 8 | 2.5 | 0.47 | 8 | 2.5 | 0.51 | 8.5 | 3.2 | 0.65 |
| TdV | 9 | 8 | 4.5 | 0.88 | 7 | 3.7 | 0.78 | 7 | 4.1 | 0.85 | 6 | 2.7 | 0.49 | 4 | 2.0 | 0.31 | 4 | 2.5 | 0.52 | 6.0 | 3.3 | 0.64 |
| LdR | 4 | 5 | 4.4 | 0.88 | 3 | 3.0 | 0.75 | 4 | 3.3 | 0.67 | 3 | 2.7 | 0.58 | 2 | 2.0 | 0.42 | 2 | 2.0 | 0.33 | 3.2 | 2.9 | 0.60 |
| Fon | 7 | 9 | 5.2 | 0.94 | 2 | 1.9 | 0.43 | 7 | 4.6 | 0.88 | 4 | 2.6 | 0.50 | 3 | 2.1 | 0.38 | 3 | 2.2 | 0.38 | 4.7 | 3.1 | 0.58 |
| CdS | 1 | - | - | - | 1 | - | - | 2 | - | - | 2 | - | - | 1 | - | - | 2 | - | - | 1.6 | - | - |
| VP | 1 | 2 | - | - | 2 | - | - | 2 | - | - | 1 | - | - | 2 | - | - | 2 | - | - | 1.8 | - | - |
| Mtg | 48 | 14 | 4.3 | 0.86 | 9 | 3.8 | 0.79 | 11 | 3.9 | 0.80 | 7 | 2.8 | 0.60 | 8 | 2.5 | 0.49 | 8 | 2.9 | 0.62 | 9.5 | 3.4 | 0.69 |
| AP | 16 | 15 | 4.9 | 0.92 | 7 | 3.7 | 0.79 | 8 | 3.6 | 0.77 | 4 | 2.6 | 0.59 | 6 | 2.3 | 0.39 | 7 | 3.2 | 0.65 | 7.8 | 3.4 | 0.68 |
| Thu | 8 | 8 | 4.7 | 0.89 | 5 | 3.2 | 0.65 | 5 | 3.8 | 0.83 | 5 | 2.8 | 0.54 | 2 | 1.4 | 0.13 | 4 | 2.4 | 0.44 | 4.8 | 3.0 | 0.58 |
| Ces | 9 | 7 | 4.7 | 0.88 | 8 | 3.7 | 0.74 | 5 | 4.0 | 0.83 | 3 | 2.0 | 0.34 | 4 | 2.4 | 0.47 | 3 | 2.2 | 0.43 | 5.0 | 3.1 | 0.62 |
| FD | 5 | 4 | 3.2 | 0.70 | 4 | 3.1 | 0.68 | 4 | 3.4 | 0.78 | 4 | 3.4 | 0.83 | 3 | 2.8 | 0.67 | 3 | 2.7 | 0.60 | 3.7 | 3.1 | 0.71 |
| Rim | 11 | 5 | 3.3 | 0.74 | 4 | 2.5 | 0.50 | 6 | 3.4 | 0.72 | 4 | 2.8 | 0.63 | 3 | 2.2 | 0.51 | 5 | 3.5 | 0.76 | 4.5 | 2.9 | 0.64 |
| Fob | 2 | 2 | - | - | 2 | - | - | 3 | - | - | 2 | - | - | 1 | - | - | 2 | - | - | 2.0 | - | - |
| Rima | 2 | 2 | - | - | 2 | - | - | 2 | - | - | 1 | - | - | 2 | - | - | 2 | - | - | 1.8 | - | - |
| Car | 2 | 4 | - | - | 3 | - | - | 3 | - | - | 3 | - | - | 2 | - | - | 3 | - | - | 3.0 | - | - |
| AdM | 2 | 2 | - | - | 2 | - | - | 3 | - | - | 4 | - | - | 2 | - | - | 2 | - | - | 2.5 | - | - |
| SA | 9 | 7 | 4.2 | 0.86 | 7 | 3.8 | 0.80 | 6 | 4.0 | 0.84 | 4 | 2.7 | 0.60 | 3 | 2.5 | 0.58 | 4 | 2.7 | 0.55 | 5.2 | 3.3 | 0.71 |
| Carì | 21 | 7 | 3.4 | 0.71 | 6 | 3.3 | 0.72 | 7 | 3.5 | 0.75 | 6 | 2.9 | 0.62 | 7 | 3.3 | 0.68 | 5 | 3.1 | 0.66 | 6.3 | 3.2 | 0.69 |
| Tri | 11 | 6 | 3.9 | 0.83 | 6 | 3.3 | 0.71 | 6 | 3.1 | 0.65 | 5 | 2.8 | 0.59 | 3 | 1.9 | 0.32 | 6 | 3.5 | 0.74 | 5.3 | 3.1 | 0.64 |
| Cam | 12 | 9 | 4.3 | 0.85 | 5 | 2.7 | 0.60 | 5 | 3.1 | 0.70 | 6 | 3.3 | 0.71 | 5 | 2.4 | 0.44 | 6 | 3.5 | 0.77 | 6.0 | 3.2 | 0.68 |
| Total/Average |  | 17 | 4.2 | 0.85 | 15 | 3.2 | 0.71 | 14 | 3.7 | 0.80 | 12 | 2.8 | 0.59 | 17 | 2.3 | 0.52 | 13 | 2.80 | 0.62 | 4.7 | 3.2 | 0.65 |
| ***P. atrimandibularis*** | |  |  |  |  |  |  |  |  |  |  |  |  |  |  |  |  |  |  |  |  |  |
| VCun | 11 | 6 | 3.8 | 0.80 | 6 | 2.9 | 0.60 | 1 | - | - | 5 | 3.2 | 0.71 | 8 | 3.7 | 0.74 | 6 | 2.9 | 0.58 | 6.2 | 3.3 | 0.69 |
| VSus | 18 | 7 | 3.8 | 0.80 | 7 | 3.6 | 0.76 | 1 | - | - | 5 | 3.2 | 0.72 | 7 | 4.0 | 0.84 | 4 | 2.4 | 0.50 | 6.0 | 3.4 | 0.72 |
| VTel | 4 | 3 | 2.8 | 0.67 | 5 | 4.2 | 0.88 | 1 | - | - | 3 | 2.7 | 0.58 | 4 | 3.3 | 0.67 | 3 | 3.0 | 0.83 | 3.6 | 3.2 | 0.73 |
| Total/Average |  | 7 | 3.5 | 0.75 | 7 | 3.6 | 0.74 | 1 | - | - | 7 | 3.0 | 0.67 | 11 | 3.7 | 0.75 | 7 | 2.8 | 0.64 |  | 3.3 | 0.71 |

**Table S3.** Hardy-Weinberg equilibrium and inbreeding coefficient (*F*_IS_) for each DNA microsatellite locus in each population. *P* (SE) values are probabilities for loci to be in Hardy-Weinberg equilibrium, based on exact tests (Guo and Thompson, 1992). Only Pdom1 in Montgenèvre population of *P. biglumis* deviated significantly from Hardy-Weinberg equilibrium after a standard Bonferroni correction (highlighted, critical value for rejecting the null hypothesis α = 0.0005). Populations are coded as in Table S1.

|  | Pdom1 | | Pdom7 | | Pdom25 | | Pdom140 | | Pdom121 | | Pdom139 | |
| --- | --- | --- | --- | --- | --- | --- | --- | --- | --- | --- | --- | --- |
|  | P (SE) | *F*_IS_ | P (SE) | *F*_IS_ | P (SE) | *F*_IS_ | P (SE) | *F*_IS_ | P (SE) | *F*_IS_ | P (SE) | *F*_IS_ |
| ***P. biglumis*** | |  |  |  |  |  |  |  |  |  |  |  |
| Fer | 0.648 (0.025) | 0.039 | 0.907 (0.010) | 0.015 | 0.775 (0.016) | -0.056 | 0.858 (0.009) | -0.132 | 0.459 (0.029) | -0.133 | 0.048 (0.009) | 0.151 |
| TdV | 0.487 (0.021) | -0.000 | 0.648 (0.017) | 0.143 | 0.542 (0.014) | 0.082 | 1 (0.000) | -0.143 | 1 (0.000) | -0.067 | 1 (0.000) | -0.280 |
| LdR | 1 (0.000) | -0.143 | 1 (0.000) | 0.111 | 0.427 (0.009) | 0.250 | 1 (0.000) | -0.286 | 1 (0.000) | -0.200 | - | - |
| Fon | 0.448 (0.023) | -0.063 | 1 (0.000) | -0.333 | 1 (0.000) | -0.132 | 0.167 (0.006) | 0.143 | 1 (0.000) | -0.125 | 1 (0.000) | -0.067 |
| CdS |  |  |  |  |  |  |  |  |  |  |  |  |
| VP |  |  |  |  |  |  |  |  |  |  |  |  |
| Mtg | 0.000 (0.000) | 0.092 | 0.557 (0.021) | 0.028 | 0.326 (0.025) | 0.018 | 0.028 (0.006) | 0.151 | 0.003 (0.002) | 0.330 | 0.225 (0.021) | 0.022 |
| AP | 0.481 (0.037) | 0.043 | 0.013 (0.004) | -0.029 | 0.866 (0.012) | -0.144 | 0.105 (0.004) | -0.068 | 1 (0.000) | -0.123 | 0.570 (0.025) | 0.014 |
| Thu | 0.788 (0.016) | -0.120 | 0.858 (0.007) | -0.343 | 0.660 (0.008) | 0.097 | 0.564 (0.015) | 0.067 | - | - | 1 (0.000) | -0.143 |
| Ces | 0.620 (0.017) | 0.152 | 0.750 (0.020) | -0.208 | 0.846 (0.006) | -0.204 | 1 (0.000) | -0.105 | 0.519 (0.011) | 0.059 | 0.378 (0.006) | 0.125 |
| FD | 1 (0.000) | -0.143 | 0.237 (0.007) | 0.407 | 1 (0.000) | -0.032 | 0.188 (0.005) | 0.515 | 1 (0.000) | -0.125 | 1 (0.000) | -0.333 |
| Rim | 0.787 (0.007) | 0.012 | 0.647 (0.009) | -0.000 | 0.394 (0.016) | -0.013 | 0.791 (0.005) | -0.151 | 1 (0.000) | 0.022 | 0.766 (0.009) | 0.063 |
| Fob | 1 0.000) | -1.000 | - | - | 1 (0.000) | -0.333 | - | - | - | - | 1 (0.000) | -1.000 |
| Rima | 1 (0.000) | -1.000 | 1 (0.000) | -1.000 | 1 (0.000) | -1.000 | - | - |  | -- | 1 (0.000) | -1.000 |
| Car | 1 (0.000) | 0.000 | - | - | 0.334 (0.005) | 0.500 | 1 (0.000) | -0.333 | 1 (0.000) | -1.000 | 1 (0.000) | -0.333 |
| AdM | 0.331 (0.002) | 1.000 | - | - | 1 (0.000) | -0.333 | 1 (0.000) | 0.000 | 1 (0.000) | -1.000 | - | - |
| SA | 0.439 (0.015) | 0.226 | 0.547 (0.017) | 0.165 | 0.663 (0.011) | 0.2067 | 0.419 (0.010) | 0.264 | 0.027 (0.002) | 0.429 | 0.712 (0.009) | -0.013 |
| Cari | 0.822 (0.011) | -0.012 | 0.590 (0.013) | 0.005 | 0.588 (0.017) | 0.114 | 0.133 (0.012) | 0.072 | 0.739 (0.018) | -0.035 | 0.884 (0.006) | -0.131 |
| Tri | 0.142 (0.009) | 0.006 | 0.052 (0.006) | 0.108 | 0.414 (0.018) | 0.155 | 0.250 (0.010) | 0.225 | 1 (0.000) | -0.127 | 0.126 (0.013) | 0.188 |
| Cam | 0.854 (0.014) | 0.022 | 0.435 (0.013) | 0.165 | 0.258 (0.008) | 0.163 | 0.570 (0.015) | -0.059 | 1 (0.000) | -0.148 | 0.769 (0.009) | 0.087 |
| ***P. atrimandibularis*** | |  |  |  |  |  |  |  |  |  |  |  |
| VCun | 0.779 (0.009) | 0.125 | 0.025 (0.005) | 0.237 | - | - | 0.462 (0.012) | -0.154 | 0.951 (0.008) | -0.104 | 0.654 (0.017) | -0.039 |
| VSus | 0.352 (0.015) | -0.187 | 0.270 (0.015) | 0.126 | - |  | 0.062 (0.005) | 0.203 | 0.987 (0.002) | 0.0695 | 1.000 (0.000) | -0.135 |
| VTel | 1.000 (0.000) | -0.125 | 0.659 (0.011) | 0.143 | - | - | 1.000 (0.000) | -0.286 | 0.438 (0.010) | 0.250 | 0.195 (0.004) | 0.600 |

**Table S4.** Pairwise *F*_ST_ estimates (bottom diagonal) in *P. biglumis* and their associated probabilities for *F*_ST_ to be significantly large than zero (top diagonal) in DNA microsatellites; calculated for populations with n≥2; estimates significantly larger than zero (P<0.05) also highlighted. Populations are coded as in Table S1.

Fe TdV LdR Fon Mtg AP Thu Ces FD Rim Fob Rima Car AdM SA Cari Tri Cam

Fe 0.532 0.775 0.640 0.640 0.432 0.784 0.964 0.117 0.054 0.099 0.108 0.306 0.162 0.901 0.000 0.054 0.360

TdV -0.004 0.622 0.820 0.342 0.775 0.982 0.955 0.000 0.099 0.063 0.351 0.180 0.126 0.703 0.045 0.333 0.460

LdR -0.022 -0.015 0.180 0.883 0.667 0.577 0.523 0.234 0.063 0.423 0.252 0.595 0.243 0.856 0.108 0.189 0.559

Fon -0.009 -0.019 0.018 0.198 0.342 0.847 0.937 0.018 0.009 0.162 0.063 0.126 0.180 0.820 0.045 0.117 0.279

Mtg -0.003 0.003 -0.027 0.018 0.748 0.468 0.523 0.090 0.027 0.225 0.045 0.405 0.630 0.748 0.000 0.207 0.577

AP -0.000 -0.014 -0.018 0.000 -0.004 0.387 0.595 0.018 0.072 0.351 0.072 0.144 0.063 0.703 0.000 0.171 0.234

Thu -0.014 -0.035 -0.016 -0.027 0.003 -0.005 0.955 0.018 0.054 0.027 0.189 0.243 0.126 0.621 0.018 0.486 0.432

Ces -0.022 -0.030 -0.021 -0.036 -0.001 -0.011 -0.036 0.018 0.207 0.081 0.189 0.072 0.514 0.982 0.045 0.243 0.414

FD 0.021 0.080 0.022 0.070 0.030 0.056 0.067 0.047 0.541 0.153 0.117 0.883 0.712 0.758 0.099 0.099 0.252

Rim 0.014 0.028 0.038 0.031 0.021 0.029 0.025 0.005 -0.010 0.018 0.081 0.487 0.793 0.541 0.000 0.360 0.450

Fob 0.051 0.084 0.006 0.082 0.044 -0.008 0.116 0.090 0.098 0.105 0.387 0.640 0.306 0.559 0.036 0.099 0.243

Rima 0.046 0.008 0.017 0.055 0.079 0.074 0.035 0.028 0.124 0.088 0.259 0.378 0.333 0.432 0.018 0.072 0.027

Car 0.009 0.055 -0.018 0.044 0.019 0.036 0.045 0.055 -0.075 -0.018 0.026 0.062 0.991 0.758 0.036 0.225 0.630

AdM 0.025 0.055 0.042 0.022 0.041 0.042 0.065 0.020 -0.045 -0.035 0.051 0.086 -0.071 0.784 0.090 0.225 0.333

SA -0.014 -0.006 -0.030 -0.015 -0.010 -0.007 -0.004 -0.032 -0.008 -0.001 0.015 0.049 0.002 -0.014 0.586 0.739 0.532

Carì 0.025 0.021 0.038 0.030 0.029 0.032 0.030 0.020 0.032 0.052 0.089 0.116 0.072 0.075 -0.002 0.036 0.000

Tri 0.014 0.006 0.028 0.019 0.011 0.007 0.003 0.007 0.043 0.002 0.096 0.126 0.044 0.060 -0.006 0.032 0.793

Cam 0.002 0.000 -0.018 0.008 -0.003 0.004 -0.001 0.000 0.016 0.002 0.032 0.089 -0.010 0.025 0.000 0.036 -0.012

**Table S5.** Pairwise *Φ*_ST_ estimates (bottom diagonal) in *P. biglumis* and their associated probabilities for *Φ*_ST_ >0 (top diagonal) in AFLP markers; calculated for populations with n≥2; estimates significantly larger than zero (P<0.05) also highlighted. Populations are coded as in Table S1.

Fe TdV LdR Fon Mtg AP Thu Ces FD Rim Fob Rima Car AdM SA Cari Tri Cam

Fe 0.270 0.333 0.940 0.126 0.189 0.450 0.414 0.135 0.306 0.387 0.378 0.405 0.279 0.072 0.288 0.450 0.369

TdV 0.006 0.396 0.991 0.297 0.595 0.252 0.721 0.991 0.991 0.991 0.991 0.991 0.991 0.991 0.531 0.703 0.991

LdR 0.006 0.007 0.703 0.532 0.261 0.279 0.991 0.477 0.441 0.622 0.712 0.676 0.595 0.153 0.505 0.477 0.514

Fon -0.056 -0.029 -0.009 0.667 0.793 0.766 0.784 0.991 0.991 0.991 0.991 0.991 0.991 0.991 0.829 0.946 0.991

Mtg 0.005 0.002 -0.007 -0.025 0.333 0.289 0.306 0.154 0.559 0.252 0.297 0.423 0.532 0.091 0.225 0.144 0.252

AP 0.005 -0.001 0.009 -0.017 0.002 0.459 0.297 0.234 0.450 0.486 0.315 0.396 0.613 0.189 0.703 0.559 0.486

Thu 0.004 0.008 0.025 -0.024 0.000 0.008 0.288 0.252 0.405 0.369 0.342 0.414 0.486 0.000 0.450 0.198 0.198

Ces 0.003 0.002 -0.046 -0.012 0.003 0.013 0.022 0.523 0.486 0.820 0.775 0.739 0.820 0.423 0.252 0.432 0.414

FD 0.027 0.000 0.025 0.000 0.028 0.014 0.039 0.015 0.514 0.991 0.991 0.991 0.991 0.991 0.342 0.577 0.991

Rime 0.002 -0.011 0.007 -0.062 -0.007 -0.001 -0.002 0.003 0.010 0.820 0.586 0.730 0.730 0.685 0.676 0.550 0.658

Fob 0.034 0.000 0.034 0.000 0.035 0.018 0.051 0.020 0.000 0.013 0.991 0.991 0.991 0.991 0.496 0.739 0.991

Rima 0.034 0.000 0.034 0.000 0.035 0.018 0.051 0.020 -0.111 0.013 0.000 0.991 0.991 0.991 0.559 0.766 0.991

Car 0.034 0.000 0.034 0.000 0.035 0.018 0.051 0.020 0.000 0.013 0.000 0.000 0.991 0.991 0.459 0.739 0.991

AdM 0.034 0.000 0.034 0.000 0.035 0.018 0.051 0.020 0.000 0.013 -0.333 0.000 0.000 0.991 0.514 0.667 0.991

SA 0.025 0.000 0.023 0.000 0.026 0.013 0.035 0.020 0.000 -0.001 -0.059 0.000 0.000 -0.059 0.153 0.459 0.991

Carì 0.006 -0.003 0.001 -0.022 0.004 -0.009 0.004 0.010 0.014 -0.005 0.018 0.018 0.018 0.018 0.013 0.802 0.739

Tri -0.001 -0.001 0.006 -0.037 0.009 -0.007 0.022 0.013 0.010 0.002 0.013 0.013 0.013 0.013 0.009 -0.014 0.667

Cam -0.002 -0.009 -0.002 -0.043 0.004 -0.003 0.014 0.004 0.000 -0.006 0.000 0.000 0.000 0.000 0.000 -0.008 -0.006

**Table S6.** Pairwise *Φ*_ST_ estimates (bottom diagonal) in *P. biglumis* and their associated probabilities for *Φ*_ST_ >0 (top diagonal) in mtDNA markers; calculated for populations with n≥2; estimates significantly larger than zero (P<0.05) also highlighted. Populations are coded as in Table S1.

Fer TdV LdR Fon Mtg AP Thu Ces FD Rim Fob Rima Car AdM SA Cari Tri Cam

Fer 0.712 0.991 0.991 0.000 0.009 0.108 0.054 0.018 0.000 0.108 0.712 0.009 0.171 0.009 0.000 0.000 0.000

TdV -0.035 0.991 0.991 0.108 0.063 0.541 0.180 0.045 0.000 0.496 0.991 0.045 0.441 0.027 0.000 0.000 0.000

LdR -0.119 -0.057 0.991 0.126 0.234 0.640 0.279 0.108 0.036 0.225 0.991 0.045 0.162 0.018 0.000 0.000 0.000

Fon -0.096 -0.113 -0.108 0.072 0.099 0.315 0.243 0.009 0.018 0.225 0.991 0.009 0.288 0.036 0.000 0.000 0.000

Mtg 0.172 0.085 0.103 0.130 0.883 0.874 0.991 0.000 0.000 0.198 0.387 0.054 0.180 0.009 0.000 0.000 0.000

AP 0.212 0.133 0.117 0.191 -0.059 0.721 0.991 0.027 0.000 0.135 0.333 0.081 0.054 0.000 0.000 0.000 0.000

Thu 0.147 0.008 0.111 0.061 -0.109 -0.071 0.991 0.153 0.009 0.414 0.784 0.135 0.477 0.108 0.009 0.009 0.000

Ces 0.188 0.068 0.120 0.131 -0.123 -0.124 -0.256 0.126 0.009 0.225 0.550 0.153 0.225 0.018 0.018 0.027 0.000

FD 0.403 0.255 0.429 0.376 0.240 0.288 0.240 0.253 0.027 0.541 0.532 0.180 0.694 0.676 0.000 0.018 0.000

Rim 0.385 0.259 0.400 0.350 0.249 0.297 0.265 0.273 0.333 0.162 0.126 0.099 0.072 0.144 0.018 0.045 0.009

Fob 0.318 0.100 0.344 0.226 0.124 0.202 0.111 0.139 0.045 0.186 0.991 0.991 0.991 0.991 0.090 0.072 0.027

Rima -0.034 -0.137 -0.081 -0.091 0.061 0.108 -0.018 0.041 0.045 0.247 -0.333 0.315 0.991 0.838 0.099 0.081 0.009

Car 0.492 0.315 0.642 0.455 0.319 0.417 0.420 0.416 0.571 0.404 0.000 0.500 0.991 0.500 0.009 0.018 0.018

AdM 0.318 0.100 0.344 0.226 0.124 0.202 0.111 0.139 0.045 0.186 -1.000 -0.333 0.000 0.991 0.099 0.099 0.027

SA 0.297 0.141 0.291 0.224 0.155 0.208 0.148 0.165 -0.010 0.109 -0.371 -0.091 0.082 -0.371 0.027 0.009 0.000

Carì 0.427 0.339 0.436 0.424 0.267 0.298 0.316 0.300 0.410 0.202 0.348 0.348 0.536 0.348 0.304 0.541 0.135

Tri 0.536 0.453 0.634 0.567 0.400 0.464 0.503 0.491 0.597 0.369 0.565 0.565 0.765 0.565 0.440 -0.007 0.423

Cam 0.669 0.642 0.863 0.771 0.539 0.633 0.758 0.723 0.861 0.598 0.878 0.878 1.000 0.878 0.659 0.135 0.054

**Table S7.** Pairwise distances between study populations (in km). Populations are coded as in Table S1.

Fer TdV LdR Fon Mtg AP Thu Ces FD Rim Fob Rima Car AdM SA Cari Tri

Fer

TdV 32

LdR 38 6

Fon 26 43 47

Mtgy 64 91 96 51

AP 57 80 85 38 17

Thu 60 86 91 44 8 9

Ces 66 92 97 50 6 14 6

FD 163 171 173 137 126 117 121 120

Rim 196 203 204 170 159 151 155 153 34

Fob 194 201 203 168 156 148 152 150 31 4

Rima 186 195 197 160 145 138 142 140 24 17 17

Car 191 199 200 165 150 143 147 145 27 12 8 6

AdM 175 182 183 149 139 131 135 134 14 21 20 18 19

SA 176 185 187 150 136 128 132 130 14 24 21 10 15 15

Carì 276 281 282 250 238 231 235 232 114 80 83 93 88 101 103

Tri 232 317 316 198 304 292 298 298 184 156 160 173 167 170 180 110

Cam 320 313 312 295 301 189 295 296 182 155 160 172 167 168 179 111 6

**Table S8.** Pairwise *F*_ST_ estimates (bottom diagonal) in *P. atrimandibularis* and their associated probabilities for *F*_ST_>0 (top diagonal) in DNA microsatellites; calculated for regions with n≥2; estimates significantly larger than zero (P>0.05) also highlighted. Regions are coded as in Table S1.

VCun VSus VTel

VCun 0.000 0.009

VSus 0.075 0.054

VTel 0.175 0.062

**Table S9** Pairwise *Φ*_ST_ estimates (bottom diagonal) in *P. atrimandibularis* and their associated probabilities for *Φ*_ST_ >0 (top diagonal) in AFLP markers. Regions are coded as in Table S1.

VCun VSus VTel

VCun 0.090 0.514

VSus 0.025 0.441

VTel 0.020 0.017

**Table S10** Pairwise *Φ*_ST_ estimates (bottom diagonal) in *P. atrimandibularis* and their associated probabilities for *Φ*_ST_ >0 (top diagonal) in mtDNA markers; estimates significantly larger than zero (P>0.05) also highlighted. Regions are coded as in Table S1.

VCun VSus VTel

VCun 0.081 0.000

VSus 0.204 0.000

VTel 0.511 0.776
